# Percutaneous Treatment of Type A Aortic Dissection Using Atrial Septal Defect Occlusion Device

**DOI:** 10.3390/jcdd13020072

**Published:** 2026-02-02

**Authors:** Georgiana Pintea Bentea, Marielle Morissens, Pierre-Emmanuel Massart, Jose Castro Rodriguez

**Affiliations:** 1 Department of Cardiology, CHU Namur Sainte Elisabeth, 5000 Namur, Belgium; 2 Department of Cardiology, CHU Brugmann, 1020 Brussels, Belgium

**Keywords:** type A aortic dissection, percutaneous treatment

## Abstract

Endovascular techniques are commonly employed for type B aortic dissections and are rarely reported for type A dissections. We present the case of a 78-year-old female diagnosed with a type A aortic dissection, with coronary arteries and supra-aortic vessels perfused from the true lumen and no significant aortic valve dysfunction. Given her recent cardiovascular surgery, the anticipated prolonged recovery, and multiple comorbidities, a percutaneous approach was preferred, with classical surgery available as stand by. The patient underwent endovascular treatment using two self-expandable, double-disc atrial septal defect occlusion devices. Intracardiac echocardiography facilitated device deployment, offering superior visualization compared with transesophageal echocardiography, which can be partially obscured by the left pulmonary artery. To our knowledge, the false lumen-to-true lumen approach in percutaneous management of type A aortic dissection has not been previously described.

## 1. Introduction

Endovascular techniques are used in treating mostly type B aortic dissection [1] and rarely reported in the literature for treating type A aortic dissection (TAAD) [2]. Emergent open surgical repair remains the standard treatment for TAAD, but perioperative mortality is high, and long-term survival is limited [3]. Approximately 25% of patients are ineligible for surgery due to comorbidities, advanced age, or refusal, with medical management carrying an in-hospital mortality of ~60%. Thoracic endovascular aortic repair (TEVAR) has been reported as a last-resort option in high-risk patients, yet evidence is limited to small case series, and no device is approved for endovascular TAAD repair. Advances in parallel stents, windowing techniques, and branched grafts have extended endovascular repair to previously inaccessible regions, including the aortic arch and descending aorta [4]. Endovascular treatment is increasingly considered for fragile patients, but the aortic root–ascending aorta segment—the final 5 cm of repair—remains challenging. Existing ascending aorta devices require proximal anchoring ≥2 cm from the coronary ostia, restricting their use to lesions sparing the aortic root, which is involved in most TAAD, making this segment a major barrier to endovascular therapy.

## 2. Case Presentation

**Figure 1:**
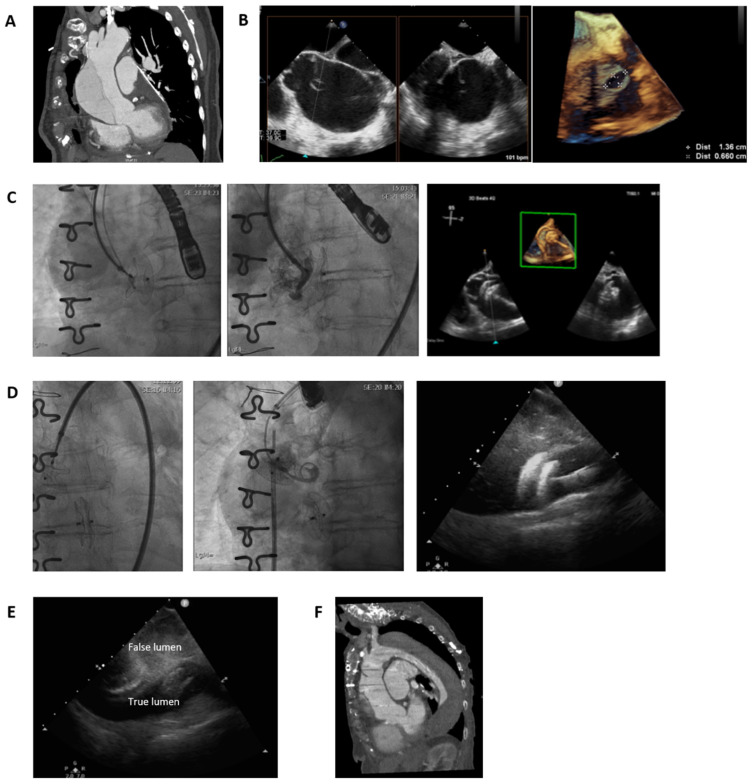
Percutaneous treatment of type A aortic dissection using atrial septal defect occlusion device: (**A**) Thoracic CT scan demonstrating a type A aortic dissection along the left lateral aspect of the aorta, with coronary arteries and supra-aortic vessels perfused from the true lumen. (**B**) Proximal entry site of the aortic dissection located 2 cm above the aortic valve, measuring 1.3 × 0.7 cm. (**C**) Occlusion of the proximal dissection entry site using a self-expandable, double-disc, 20 mm atrial septal defect occlusion, positioned from the false lumen to the true lumen. (**D**) Occlusion of the residual aortic arch dissection entry site using a self-expandable, double-disc, 18 mm atrial septal defect occlusion device. (**E**) Intracardiac echocardiography demonstrating precise and stable deployment of both occlusion devices, with immediate stasis observed in the false lumen. (**F**) Two-year follow-up CT scan showing complete thrombosis of the false lumen.

A 78-year-old female patient was diagnosed with a main pulmonary artery aneurysm and pulmonary valve regurgitation in the context of progressive dyspnoea and followed up for 6 years until the pulmonary artery aneurysm reached 80 mm and the debilitating dyspnoea required surgical treatment. She underwent pulmonary artery plasty and a pulmonary valve homograft. The anatomopathological examination of the pulmonary artery showed giant cell arteritis. However, the patient was never diagnosed nor treated with immunosuppressive therapy for this disease. Her comorbidities included chronic obstructive pulmonary disease (COPD), and the cardiovascular risk factors included high blood pressure and previous smoking. She had a normal renal function and no other significant comorbidities. The 3-month follow-up thoracic CT-scan showed a type A aortic dissection (TAAD), on the left side of the aorta, with coronary arteries and supra-aortic vessels perfused from the aortic true lumen (Figure 1A). The patient was completely asymptomatic, and there was no significant dysfunction of the aortic valve. The TAAD entry point was 2 cm supra-valvular and presented a breach in the intima that could correspond to the aortic cannulation site from the previous cardiac surgery (Figure 1B). The dimensions of the false lumen at its biggest diameter reached 75% of the diameter of the aorta. Transoesophageal echocardiography (TEE) demonstrated the same entry site and revealed free flow from the true lumen to the false lumen on colour Doppler imaging. Due to the recent cardiovascular surgery, the difficult and lengthy recovery, a frailty Identification of Seniors at Risk (ISAR) score superior to 2, and the multiple comorbidities, a percutaneous approach of the TAAD was preferred with classical surgery stand by after multidisciplinary discussion in the Heart Team. This was further compounded by the patient’s expressed desire to avoid another open heart surgery with cardiopulmonary bypass. Conservative management was also deemed suboptimal due to the risk of aneurysm progression and potential rupture. After careful evaluation, the interventional approach was selected as the safest and most effective strategy, balancing procedural risk with long-term outcomes. The percutaneous procedure was explained at length to the patient in the presence of her family, including its novelty, potential complications and uncertain likelihood of success. We used a femoral artery approach and with intraprocedural TEE identified another entry site of the aortic dissection at the level of the aortic arch situated 2 cm proximal to the brachio-cephalic artery. The maximal diameter of the proximal entry site was 1.3 × 0.7 cm. A sizing balloon was not used to avoid the risk of further tearing the defect, despite the potential device size-mismatch related complications. Instead, TEE was used to estimate its dimensions and guide selection of the atrial septal defect (ASD) occluder device. We proceeded to guidewire passage into the false lumen at this level and occluded the proximal dissection entry site using a self-expandable, double-disc, 20 mm atrial septal defect occlusion with a false lumen towards true lumen approach (Figure 1C). After the procedure, we observed blood stasis in the false lumen and as such considered the intervention to be successful and sufficient. In addition, TEE alone did not provide adequate visualization of the more distal entry site. However, a follow-up CT scan performed 4 months later revealed a persistent second dissection entry site at the level of the aortic arch. Therefore, occlusion of this entry site was subsequently performed. Via a femoral artery approach, we passed the guidewire into the false lumen guided by intracardiac echocardiography, its advantage over TEE being that it is not obscured by the left pulmonary artery, and we occluded the residual dissection entry site using a self-expandable, double-disc, 18 mm atrial septal defect occlusion device (Figure 1D). Intracardiac echocardiography demonstrated precise and stable deployment of both occlusion devices. Aortic stasis within the false lumen was immediately apparent during the procedure as assessed by TEE performed by a specialist in echocardiography (Figure 1E), and 2-year follow-up CT scan demonstrated complete thrombosis of the false lumen (Figure 1F). Furthermore, the patient remained asymptomatic, and there were no signs of ASD occluder device migration or erosion, nor recurrent aortic dissection, as documented by repeated CT scan and TEE.

## 3. Discussion

Acute TAAD is associated with a 50% mortality within the first 48 h if not operated on. Surgery reduces 1-month mortality from 90% to 30% [5]. In the present case, the patient was entirely asymptomatic at the time of diagnosis of TAAD and was therefore considered hemodynamically stable. The dissection was likely iatrogenic, related to aortic cannulation during prior cardiac surgery. This provided both the patient and the clinical team additional time to deliberate and evaluate alternative therapeutic options. Alternative percutaneous interventions to manage the TAAD by inducing thrombosis of the false lumen were described, employing occluder devices, vascular plugs, distal stent-grafts or coils and glue [6]. More recently, an emerging endovascular technique, the Endo-Bentall procedure, has also been described for the treatment of ascending aortic dissection in patients at high surgical risk [7]. A recent meta-analysis reported a 30-day complication rate of 7.08%, while the rate of late complications occurring beyond 30 days was 16.89%, which included endoleaks, technical failure during deployment of the graft, and post-operative stroke. Pooled 30-day and late mortality rates were 2.46% and 1.59%, respectively [3].

The novelty of our case consists of describing an endovascular treatment of type A aortic dissection using two self-expandable, double-disc atrial septal defect occlusion devices. 

The choice of ASD occlusion device should be guided by the diameter of the entry tear. Consequently, the limited range of currently available device sizes represents a major constraint for this technique. We also believe that ASD closure devices are not suitable for hemodynamically unstable patients and should be avoided if the ascending aortic dissection involves supra-aortic bifurcation.

Therapeutic anticoagulation or antiplatelet medication was not implemented in order to facilitate the formation of a thrombus in the false lumen dissection. We assessed the thrombosis risk of the prosthesis as minimal given the high flow velocities in the aorta. There is currently no available literature to support this hypothesis. As such a very close imagistic follow-up (CT scan and TEE) was implemented for the following months in order to screen for possible stroke or embolic events. This approach was preferred regardless of the potential risks, given the alternative outcome of a persistent permeable aortic false lumen.

Furthermore, we report the use of percutaneous management of aortic dissection aided by intracardiac echocardiography, its advantage over TEE being that it is not obscured by the left pulmonary artery. However, intracardiac echocardiography could not replace TEE in this type of procedure and should be used as a complementary tool [8]. The false lumen-to-true lumen approach in percutaneous treatment of TAAD was not previously described to our knowledge.

Moreover, the impact of giant cell arteritis diagnosed in this patient on the development and treatment of the aortic dissection is unclear. Previous, albeit rare, case reports have described the co-occurrence of giant cell arteritis and spontaneous aortic dissection [9,10,11,12,13], which may have contributed to the development of aortic dissection at the aortic cannulation site from the prior surgery in the present case.

## Data Availability

The data presented in this study are available upon request from the corresponding author due to privacy reasons.
